# Enhanced Fatigue Crack Detection in Complex Structure with Large Cutout Using Nonlinear Lamb Wave

**DOI:** 10.3390/s24216872

**Published:** 2024-10-26

**Authors:** Suofeng Zhang, Yuan Liu, Shenfang Yuan

**Affiliations:** Research Center of Structural Health Monitoring and Prognosis, State Key Laboratory of Mechanics and Control for Aerospace Structures, Nanjing University of Aeronautics and Astronautics, Nanjing 210016, China; suofengzhang@nuaa.edu.cn

**Keywords:** large cutout, fatigue cracks, nonlinear Lamb wave, integrated data processing, phase-velocity desynchronization

## Abstract

The large cutout structure is a key component in the bottom skin of an airplane wing, and is susceptible to developing fatigue cracks under service loads. Early fatigue crack detection is crucial to ensure structural safety and reduce maintenance costs. Nonlinear Lamb wave techniques show significant potential in microcrack monitoring. However, nonlinear components are often relatively weak. In addition, a large cutout structure introduces complex boundary conditions for Lamb wave propagation, making nonlinear Lamb wave monitoring more challenging. This article proposes an integrated data processing method, combining phase inversion with continuous wavelet transform (CWT) to enhance crack detection in complex structures, with phase-velocity desynchronization adopted to suppress the material nonlinearity. Experiments on a large cutout aluminum alloy plate with thickness variations were conducted to validate the proposed method, and the results demonstrated its effectiveness in detecting fatigue cracks. Furthermore, this study found that nonlinear components are more effective than linear components in monitoring closed cracks.

## 1. Introduction

In aircraft structures, cutouts are commonly used as access ports for mechanical and electrical systems, for example, there are large cutouts provided in the bottom skin of the wing to allow entry into the airplane fuel tanks for inspection or component maintenance [1]. Fatigue cracks, which can initiate from the cutouts due to stress concentration under long-term cyclic loads, are one of the main causes of aircraft structure failure. Thus, it is crucial to detect the initiation of fatigue cracks and to monitor their progression regularly or continuously.

Lamb waves have been widely used in fatigue crack detection and the monitoring of plate-like structures due to their long-distance propagation and high efficiency [2,3,4]. The principle of Lamb wave-based assessment is that, when interacting with fatigue cracks, the propagation characteristics of the Lamb waves will be modulated and changed to a certain extent. This interaction induces specific wave-scattering phenomena [5,6] such as wave reflection, transmission, mode conversion, and the generation of higher-order harmonics. Therefore, by examining the changes in these wave characteristics, the extent of damage can be evaluated. Linear wave characteristics generally include time-of-flight delays, wave reflections or scattering, energy dissipation, and mode conversion, while nonlinear characteristics are mainly manifested as higher-order harmonics, sub-harmonics, direct current response effects, mixed-frequency modulation, and nonlinear resonances, etc. [7,8,9]. Both linear and nonlinear features have been extensively used for damage characterization. Compared to the linear features, the nonlinear properties of Lamb waves exhibit higher sensitivity to microscopic or closed damage, and the detectability is not restricted by the wavelength of the probing waves.

In recent years, second harmonics have been extensively explored for the detection and characterization of material nonlinearity and contact acoustic nonlinearity (CAN) [10,11,12,13]. It has been found that two necessary conditions—phase velocity synchronism and non-zero energy flux—need to be met for the generation of the cumulative second harmonic induced by material nonlinearity [14,15]. Most previous studies focused on the mode pairs (S1–S2, S1–S3, S2–S4, etc.) used to assess fatigue damage based on the criteria of cumulative second harmonic generation [16,17]. The approximate phase–velocity matching method (S0–S0) at low frequency, which shows the features of the weak dispersive, was also reported to evaluate pitting damage [18,19].

**Figure 1:**
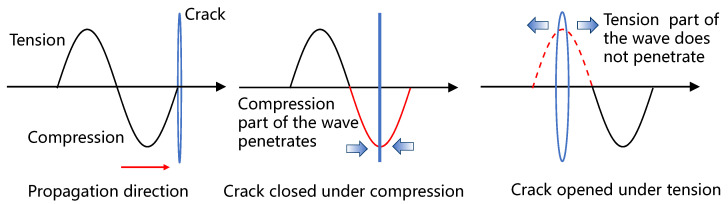
Diagram of ultrasonic waves propagating through a breathing crack.

In contrast to material nonlinearity, the CAN is one of the local nonlinearity effects based on the ‘breathing’ mechanics of contact-type damages, such as interface debonding, delamination, and fatigue cracks. When a high-amplitude ultrasonic wave encounters a “breathing” crack, the compressional part of the wave forces the crack surfaces to contact, allowing the wave to pass through, while the tensile part of the wave opens the crack further, blocking the wave transmission and causing wave scattering, as illustrated in Figure 1. The opening and closing of the crack result in stiffness changes in the crack region. When the crack is closed, the local stiffness remains unaffected, but the crack opening reduces the local stiffness, leading to the generation of nonlinear phenomena. The “breathing” crack segment can be considered a secondary sound source induced by the interaction between the “breathing” crack and the ultrasonic wave.

Wang et al. [20,21] developed an analytical model to interpret the modulation mechanism of a ‘breathing’ crack on ultrasonic guided waves, extended it to a three-dimensional regime based on wave propagation theory in three-dimensional waveguides, and defined a nonlinearity index quantitatively predicting the fatigue crack length in thin plates. Xu et al. [22] developed an analytical model based on the elastodynamic reciprocity theorem to elucidate the second harmonic generation arising from CAN at ‘breathing’ cracks and derived a closed-form solution for the magnitude of the crack-induced second harmonic. Shen and Giurgiutiu et al. [23] employed the finite element method to simulate the nonlinear interaction between Lamb waves and a ‘breathing’ crack, demonstrating that the generated second harmonic increases monotonically with crack severity. Yang et al. [24] analyzed the influence of the wave mode on the harmonic and found that the magnitude and directivity of the harmonic are related to the ratio of crack length to incident wavelength and the ratio of S0 to A0 incident Lamb wave amplitude, also showing that the S0 Lamb wave is more effective for fatigue crack detection. Lee and Lu et al. [25] utilized second harmonic generation to detect fatigue cracks in a T-shaped steel plate under vibration conditions, demonstrating the potential of using nonlinear features for the evaluation of fatigue cracks in practical structural health monitoring scenarios. Xu et al. [26] proposed a modeling framework to accurately characterize and continuously monitor fatigue cracks. The effectiveness of the framework in monitoring the initiation and progressive growth of a corner fatigue crack was validated through numerical simulations and experiments. Xu et al. [27] characterized the location and orientation of closed cracks using the second harmonic Lamb wave induced by the interaction of the temporal S0 mode Lamb wave and a closed crack, through a weighted and structured sparse decomposition algorithm. Wang and Shen et al. [28] proposed a 2D analytical framework for modeling Lamb wave generation, propagation, wave-crack linear and nonlinear interaction, and reception, adopting a physical-virtual time inversion of nonlinear Lamb waves for fatigue crack detection and quantification. The nonlinear characteristics exhibited a trend of initially increasing and then decreasing with crack growth. Compared with the contact acoustic nonlinearity arising from fatigue cracks, material nonlinearity around the cracks is marginal and contributes less to the total nonlinearity as the crack grows [29,30], but distributed material nonlinearity can occasionally make a significant contribution to the measured nonlinear components [31]. Therefore, phase velocity synchronism should be avoided to minimize the effect of material nonlinearity for reliable crack detection based on CAN. Yin et al. [32] introduced a method for microcrack localization using cross-shaped sensor clusters in a plate, where the A0–S0 wave mode pair with a distinct wave speed difference was used to minimize the cumulative growth effect of material nonlinearity. Sun et al. [33] developed a feasible method for accurately detecting and characterizing the closed-crack evolution using nonlinear Lamb waves with phase–velocity mismatching to exclude the intrinsic material nonlinearity.

Existing research mainly focuses on simple structures such as plates with uniform thickness. There has been no research based on nonlinear ultrasonic crack monitoring technology on complex structures with large openings and large thickness variations that are common on aircrafts. For these structures, the propagation of the Lamb wave is significantly influenced by complex structural features, and the received signals are often complicated due to the interaction of the crack with multiple modes, mode conversion, and reflection from boundaries. The damage information may be masked in the signals, posing additional challenges for reliable fatigue crack detection. In this paper, the feasibility of crack monitoring based on a nonlinear ultrasound is investigated for a complex structure with a varying thickness and a large cutout in the middle. An integrated signal processing method based on phase inversion technique and continuous wavelet transform (CWT) is proposed and its validity is verified experimentally. The following Section 2 introduces the methodology. Section 3 introduces the specimen and excitation frequency selection for the experimental validation, together with a numerical study to illustrate the influential mechanism of the complex geometric structure of the specimen on Lamb wave propagation. Then, the experimental study to validate the proposed method is presented in Section 4. Finally, conclusions are given in Section 5.

## 2. Methodology

This section provides a brief review of the nonlinearity of Lamb waves propagating in elastic media and proposes an integrated signal processing method based on phase inversion and a Morlet wavelet-based CWT algorithm for effective fatigue crack detection in complex structures.

### 2.1. CAN of Lamb Waves

The nonlinearities in the ultrasonic detection of fatigue cracks include [34,35,36,37]: (i) inherent material and geometric nonlinearity in the intact material, denoted as *β*_e_; (ii) plastic nonlinearity (*β*_p_) resulting from local plastic deformation near the tip of a fatigue crack; (iii) CAN from closed cracks, denoted as *β*_CAN_; and (iv) experimental equipment-induced nonlinearity (*β*_eq_). In summary, the total nonlinearity *β* can be expressed as Equation (1),

(1) $$\beta =\beta_{e}+\beta_{p}+\beta_{\mathrm{CAN}}+\beta_{\mathrm{eq}}$$

*β*_e_ and *β*_eq_ are not directly induced by the crack and need to be minimized in crack detection to achieve more accurate measurements [32,33]. In addition, studies have shown that, compared with the *β*_CAN_, *β*_p_ is marginal and contributes less to the total nonlinearity as the crack grows [34]. Therefore, *β*_CAN_ often serves as a primary index for accurately characterizing the nonlinearity generated by fatigue cracks and evaluating fatigue crack propagation.

Based on the bilinear stiffness model, Zhao et al. [38] accounted for the differing effective moduli under tensile and compressive loads for the local micro-crack structure and defined an index for contact acoustic nonlinearity (*β*_CAN_), as shown in Equation (2).

(2) $$\beta_{\mathrm{CAN}}=\frac{A_{2}}{A_{1}}$$

where *A*_1_ and *A*_2_ are the amplitudes of the fundamental wave and the second harmonic wave, respectively. This index is used in the following sections for characterizing the CAN nonlinearity and evaluating the fatigue crack.

### 2.2. Fatigue Crack Detection Based on Nonlinear Lamb Waves

#### 2.2.1. Phase Inversion Technique

There is a general consensus that the nonlinear waves induced by damage are much weaker than the primary waves [39]. Therefore, efforts have been made to suppress the fundamental frequency component and enhance the second harmonic component [40,41,42]. Phase inversion technique is a widely used method to achieve this by canceling out the odd harmonics through the addition of two 180° out-of-phase signals. Considering only the first two order components, the total wavefield of guided wave propagation can be expressed as Equation (3) [43].

(3) $$u^{0^{\circ }}=A_{1}\exp\left[j\left(kx-\omega t\right)\right]+A_{2}\exp\left[2j\left(kx-\omega t\right)\right]$$

where *u*^0°^ represents the 0° phase displacement field of guided waves, the displacement field of elastic waves in solid media can be expressed as an exponential function with the imaginary power exponent *j*. Here, *A*_1_ and *A*_2_ represent the amplitudes of the fundamental wave and the second harmonic, respectively; *k* is the wave number, *x* is the wave propagation distance, *t* denotes the time variable, and ω is the angular frequency.

With the phase in the fundamental ultrasonic wave reversed 180°, the wavefield in the waveguide changes to

(4) $$u^{180^{\circ }}=A_{1}\exp\left[j\left(kx-\omega t+\pi \right)\right]+A_{2}\exp\left[2j\left(kx-\omega t+\pi \right)\right]$$

By summing Equations (3) and (4), it is found that only the second harmonic field remains, as shown in Equation (5).

(5) $$u^{\prime }=u^{0^{\circ }}+u^{180^{\circ }}=2A_{2}\exp\left[2j\left(kx-\omega t\right)\right]$$

Phase inversion technique is demonstrated to double the second harmonic amplitude while counteracting that of the primary harmonics, effectively enhancing the efficiency of crack monitoring using *β*_CAN_ based on the second harmonic waves.

#### 2.2.2. Time–Frequency Feature Extraction Using CWT

For complex structures, the propagation of Lamb waves is often nonuniform due to the interaction between cracks and multiple modes, mode conversion, and boundary reflections. Furthermore, complex structures might have multiple potential damage locations; therefore, the source of nonlinearity is often uncertain. This brings an additional challenge in the identification and extraction of the second harmonic signal. A representation of signals in both time and frequency domains can help clearly show the arrival times of different frequency components, benefiting the identification of second harmonic signals from cracks.

The CWT [44] is an effective time–frequency analysis tool that provides an overcomplete representation of a signal, capturing transient changes and local features by continuously varying the translation and scale parameters of the wavelet function. This wavelet function can be dynamically adjusted to capture intricate signal components across various scales, overcoming the localization limitation of the Fourier transform and the fixed window size constraints of the short-time Fourier transform. For a given raw Lamb wave signal *x*(*t*), the CWT is performed as follows:

(6) $$CWT_{x}\left(a,b\right)=\frac{1}{\sqrt{a}}\int x\left(t\right)\varphi^{*}\left(\frac{t-b}{a}\right)dt,a>0$$

where *φ* represents the mother wavelet, φ^*^ is the complex conjugate of *φ*, $a\in R^{+}$ is the scaling factor that controls the wavelet’s frequency, and *b* is the translation factor that identifies its location.

The wavelet coefficients $CWT_{x}\left(a,b\right)$ are calculated by convolving the signal with a scaled and translated wavelet function, representing the similarity between the signal and the wavelet function at each scale and position. Selecting an appropriate wavelet basis function is crucial as it directly affects the accuracy of crack detection. The Morlet wavelet, a complex exponential function with a Gaussian envelope, combines the characteristics of a Gaussian window and a sine wave, offering excellent time–frequency localization [45]. Therefore, the Morlet wavelet function is selected for this study, expressed as in Equation (7) [46].

(7) $$\psi \left(t\right)=\pi^{-\frac{1}{4}}e^{i\omega_{0}t}e^{-\frac{t^{2}}{2}}$$

where $\psi \left(t\right)$ is a Morlet wavelet function, $\pi^{-1/4}$ is a constant used to normalize the amplitude of the wavelet function to ensure consistent energy across different scales, *j* is the imaginary unit, *ω*_0_ is the frequency parameter that controls the wavelet function’s frequency, and *t* is the time parameter. Accurately determining the arrival time of specific frequency components is essential for crack detection and localization.

#### 2.2.3. Integrated Data-Processing Method

**Figure 2:**
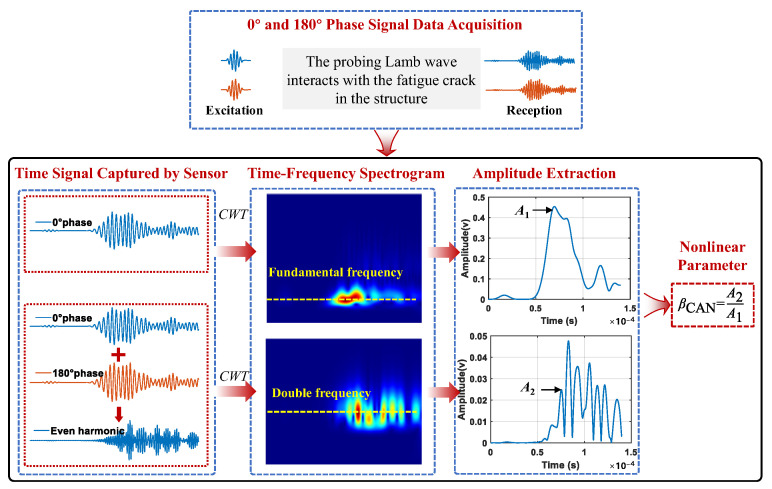
Framework of the proposed integrated data processing method.

In this section, an integrated data processing method based on phase inversion and a Morlet wavelet-based CWT is proposed for the crack monitoring of complex structures. Firstly, excitations with a phase of 0° and 180° are sent into the structure with a high power ultrasonic system, respectively, enabling the acquisition of received signals with opposite phases. Next, the time–frequency plots of the 0° phase signal and the sum of the two signals with opposite phases are extracted, where the dominant frequency components correspond to the fundamental frequency and the second harmonic, respectively. Finally, the amplitude envelopes at fundamental and double frequencies, marked by yellow dot lines, are extracted from the time–frequency plots. The envelope amplitude curves might contain multiple peaks. The peaks which arrive at the times of interest calculated based on the crack location and the wave speeds are selected. The amplitude of the select peak in the curve of the fundamental frequency is recorded as *A*_1_ and that of the double frequency is denoted as *A*_2_. The nonlinear parameter *β*_CAN_ is then calculated as $A_{2}/A_{1}$. The entire process is illustrated in Figure 2.

Due to structural complexity, multiple peaks often appear in the amplitude plots, particularly in the second harmonic, as shown in Figure 2. Therefore, to accurately calculate *β*_CAN_ induced by the crack, it is necessary to identify the peak amplitude *A*_2_ originating from the crack. If the potential location of the crack is known, the peak amplitude can be identified by the time of flight.

## 3. Specimen Description and Numerical Study

This section introduces the specimen used in the experimental validation and the selection of the excitation frequency. Additionally, a simulation study is presented to illustrate the influential mechanism of the complex geometric structure of the specimen on Lamb wave propagation, aiding in the understanding of the subsequent experimental results.

### 3.1. Specimen Description

**Figure 3:**
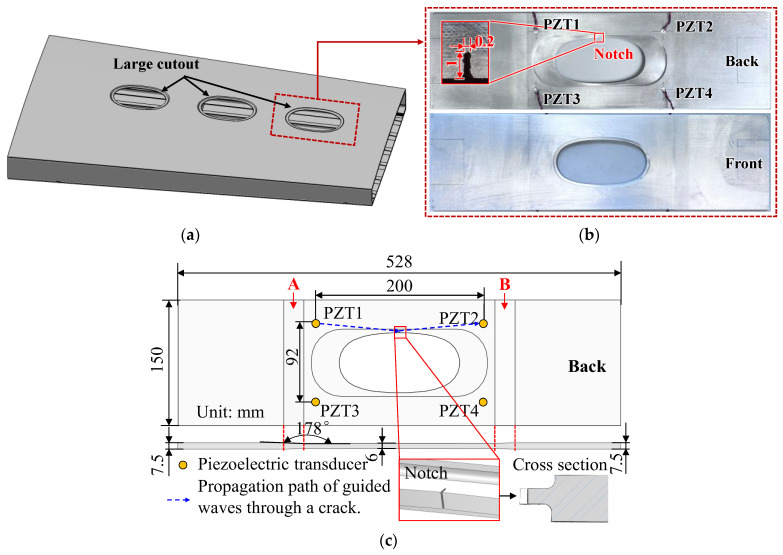
Specimen and PZT arrangement. (**a**) Bottom of wing box; (**b**) 6061-T6 aluminum specimen; and (**c**) Dimensions and PZT layouts.

The specimen, designed based on the critical fatigue part of a type of wing box and fabricated using 6061-T6 aluminum, is shown in Figure 3b. The specimen features a central elliptical hole and exhibits two regions with thickness variations: the first region includes a stepped change in thickness around the elliptical hole, as depicted in the cross-sectional inset, and the second region encompasses areas labeled A and B, where thickness also varies. A notch (1 mm in length and 0.2 mm in width) was prefabricated on one side of the large elliptical cutout to initiate a fatigue crack from the notch tip and promote unilateral fatigue crack propagation. Four identical PZTs (PSN-33, diameter: 8 mm, thickness: 0.48 mm) were bonded to the surface of the aluminum for exciting and sensing the Lamb wave and were labeled as PZT1, PZT2, PZT3, and PZT4; the positions of the PZTs are shown in Figure 3c. The PZTs were strategically arranged to minimize the complexity of the acquired guided wave signals. The sensing path PZT1–PZT2 was located near the notch, where the fatigue crack was expected to grow, while the sensing path PZT3–PZT4 was on the other side of the large cutout. The spacing between sensors PZT1 (PZT3) and PZT2 (PZT4) was set at 200 mm to separate the S0 and A0 modes as much as possible, while ensuring that regions A and B with varying thicknesses were not located on the wave path from PZT1 (PTZ3) to PZT2 (PZT4), minimizing the impact of the varying thickness regions on the received signals.

### 3.2. Mode Selection of Primary Wave

Given that the focus of this study is to measure the CAN nonlinearity caused by the “breathing” behavior of fatigue cracks, material nonlinearity should be minimized. This can be achieved by phase velocity mismatching between the fundamental and the second harmonic waves [18]. If the relative deviation of the phase velocity between the fundamental wave and second harmonic, calculated using Equation (8), is larger than the threshold value of 1%, it is considered phase velocity mismatched and the maximum cumulative propagation distance of the material nonlinearity is *L*_max_, expressed by Equation (9) [18].

(8) $$D=\frac{c_{\mathrm{ph}}^{\omega }-c_{\mathrm{ph}}^{2\omega }}{c_{\mathrm{ph}}^{\omega }}\times 100\%$$

(9) $$L_{\max}=\frac{\pi c_{\mathrm{ph}}^{2\omega }\times c_{\mathrm{ph}}^{\omega }}{2\omega \left|c_{\mathrm{ph}}^{2\omega }-c_{\mathrm{ph}}^{\omega }\right|}$$

where $c_{\mathrm{ph}}^{\omega }$ and $c_{\mathrm{ph}}^{2\omega }$ refer to phase velocities of the fundamental wave and the second harmonic, respectively.

The propagation of Lamb waves is complicated due to their inherent dispersion and multi-mode characteristics [47]. The higher the frequency, the greater the number of Lamb wave modes that can propagate at that frequency. Therefore, the frequency should be chosen following two criteria: (1) phase velocity mismatched at the fundamental and double frequencies to suppress the material nonlinearity; (2) low frequency where only a few modes exist to simplify the Lamb wave propagation. Therefore, dispersion curves are needed for frequency selection. From Figure 3c, the blue-dashed line, indicating the guided wave path of interest, clearly shows that the guided wave travels in both the 6 mm and 2 mm regions, with a relatively large portion of the 6 mm region. Therefore, the dispersion curves for a 6 mm thickness plate are used in the following context to analyze mode selection.

**Figure 4:**
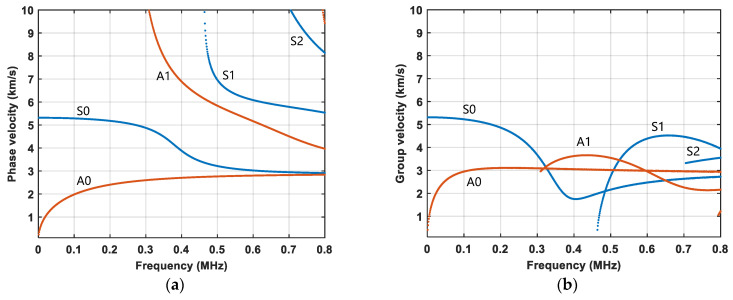
Dispersion curves for 6 mm thick 6061 aluminum plate. (**a**) Phase velocity; (**b**) Group velocity.

Figure 4 shows the dispersion curves obtained by solving the equation via numerical methods for a 6 mm thick 6061-T6 aluminum plate based on the material parameters, as listed in Table 1. Lower frequencies are considered since there are less modes at low excitation frequencies. In this work, the S0 mode at 300 kHz is chosen because only the S0 and A0 modes exist at this frequency. At 600 kHz, the second harmonic includes the A0, S0, A1, and S1 modes. Table 2 shows the relative deviation of the phase velocities between the primary wave and the second harmonic at the excitation frequency of 300 kHz. The maximum cumulative propagation distance is also shown in Table 2. The relative deviations of all four mode pairs are larger than the threshold value of 1%, Consequently, the chosen S0 mode with the frequency of 300 kHz satisfies the phase–velocity mismatching condition. The mode pairs exhibit a relatively small *L*_max_ compared to the spacing between sensors, indicating that the material-induced nonlinearity minimally contributes to second harmonic generation. Consequently, the generated second harmonic primarily results from nonlinearity at the fatigue crack.

### 3.3. Numerical Study

To analyze the effect of the complex geometric features on Lamb wave propagation, this section presents comparative simulation results between the large cutout specimen and a flat plate with a uniform thickness, based on numerical simulations conducted using the COMSOL Multiphysics computational platform.

The large cutout model and the test specimen share identical geometric dimensions, as introduced in Section 3.1. PZT1 and PZT2 are positioned on the surface of the structure at their respective locations, with the piezoelectric effect integrated into the 3D simulation model. A 300 V excitation voltage, of a 5-cycle Hann-windowed tone burst with a center frequency of 300 kHz is applied on PZT1, while voltage signals are received and averaged at PZT2. The material parameters for both types of test specimens are listed in Table 1. The low-reflecting boundary condition is adopted to minimize the wave reflections at the boundaries. To improve the accuracy of the local solution and achieve error convergence, the maximum element size is 0.2 mm. A maximum time step of 8 × 10^−8^ s is used in the simulation, as it is required to be smaller than the time resolution, 1/20 *f*_ph_, to satisfy the stability criteria [48], where *f*_ph_ is the maximum frequency of the targeted Lamb mode. For comparison, the reference model of a 6 mm thick flat plate is simulated under the same conditions, including the overall dimensions and PZTs, but without thickness variations or large cutouts.

**Figure 5:**
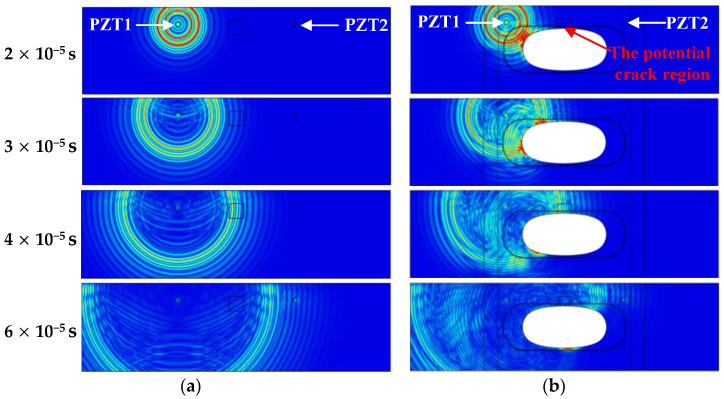
Lamb wave simulation and wave field at different time instants: (**a**) The flat plate; (**b**) the large cutout specimen.

The simulation results shown in Figure 5 highlight the differences in Lamb wave propagation between the flat plate and the large cutout specimen at four distinct time instants: 2 × 10^−5^ s, 3 × 10^−5^ s, 4 × 10^−5^ s, and 6 × 10^−5^ s. It is evident that the thickness variation and the cutout significantly affect Lamb wave propagation. Additionally, in the large cutout specimen, the potential crack is expected to initiate at the notch, in the 2 mm thick region adjacent to the cutout, while the PZTs used for wave excitation and reception are positioned in the 6 mm thick region. Therefore, with a potential crack, both the incident wave at the crack interface and the nonlinear wave generated from the “breathing” behavior interact with the reflected or scattered waves from the cutout and pass through the step area, creating a more complex wavefield compared to that in the flat plate.

## 4. Experimental Study

Experimental study was conducted to validate the proposed method. This section first presents the experimental setup for fatigue crack monitoring and then discusses the results.

### 4.1. Experimental Setup for Fatigue Crack Monitoring Tests

**Figure 6:**
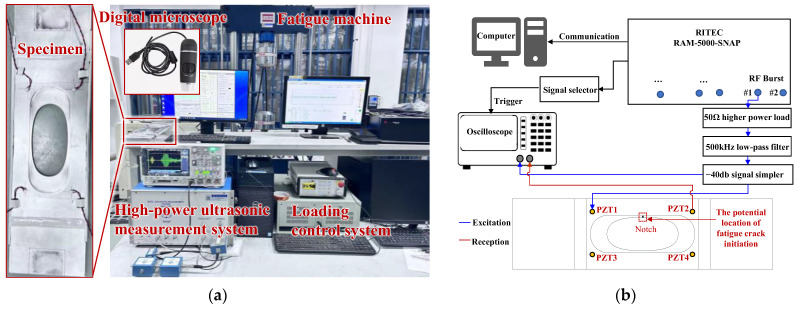
Fatigue loading system and experimental setup of ultrasonic measurement. (**a**) Experimental setup; and (**b**) Schematic diagram.

The test equipment is illustrated in Figure 6a. The SUNS 890-100 fatigue loading system was utilized to perform the fatigue test. Initially, a fatigue test was conducted with a maximum tensile load of 30 kN and a stress ratio of 0.1 to accelerate the initiation of the fatigue crack. Then, after a 0.3 mm crack was observed, the maximum load was reduced to 26 kN to slow the growth of the crack. The loading frequency was fixed at 10 Hz during the fatigue tests. During the test, the fatigue crack kept opening and closing under the load and its length was measured using a digital microscope with 220× magnification.

This specimen was loaded for a predetermined number of cycles and then unloaded to carry out the nonlinear ultrasonic measurements, after which the fatigue test was resumed and additional fatigue load cycles were applied. This process was repeated until the fatigue crack reached approximately 15 mm in length. The experimental setup for fatigue crack monitoring based on nonlinear ultrasonics is illustrated in Figure 6b. This setup includes a RITEC RAM-5000 SNAP high-power ultrasonic measurement system (RITEC, Inc., Warwick, RI, USA.), a high-power load for impedance matching, a 500 kHz low-pass filter to eliminate instrument nonlinearity, a −40 dB signal sampler, and a Keysight MSO X3014T oscilloscope (Keysight Technologies, Inc., Santa Rosa, CA, USA.). A 5-cycle Hanning-windowed tone burst at a central frequency of 300 kHz (570 V peak-to-peak voltage) was sent to drive PZT1 (PZT3). The amplitude of the excitation signal was attenuated by 40 dB before being captured by the oscilloscope. The propagating Lamb wave signals, modulated by the fatigue crack, were captured by another identical PZT2 (PZT4) using an oscilloscope at a sampling frequency of 71.4 MHz to ensure high resolution. The acquired signals were averaged 1024 times to reduce noise and measurement uncertainty from the testing system and environment.

### 4.2. Results and Discussion

#### 4.2.1. Fatigue Crack Propagation

**Figure 7:**
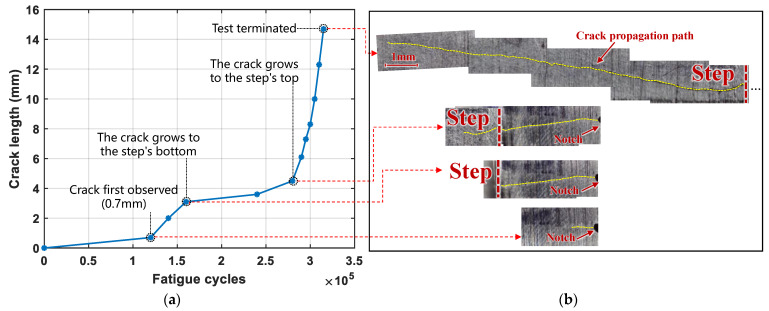
Fatigue crack growth process. (**a**) Crack length versus the fatigue cycles; (**b**) Images obtained by a digital microscope at crack lengths of 0.7 mm, 3.1 mm, 3.6 mm, and 14.7 mm, the crack length recorded at the stage of the crack initiation, propagation into the region of varying thickness, extension to the thicker region, and final appearance at the end of monitoring.

Figure 7a illustrates the crack growth progression under fatigue loading cycles. A microcrack (0.7 mm) was first observed at the notch tip of the specimen after approximately 120,000 fatigue loading cycles. Between 120,000 and 160,000 cycles, the crack propagated in the 2 mm thick region and followed a typical propagation rate. After 160,000 cycles, the rate of propagation slowed down, as the crack extended into a thicker region—the stepped thickness change area around the elliptical hole. It took approximately another 120,000 cycles for the crack to propagate from the bottom to the upper surface of the stepped area. Then, after 280,000 cycles, the crack propagation accelerated rapidly again. The test was terminated at 315,000 cycles, with the crack length growing to approximately 14.7 mm. Figure 7b shows the images obtained by a digital microscope at crack lengths of 0.7 mm, 3.1 mm, 3.6 mm, and 14.7 mm. The crack length recorded at the stage of the crack initiation, propagation into the region of varying thickness, extension to the thicker region, and final appearance at the end of monitoring. A single primary microcrack was initiated from the notch tip and propagated, remaining in a closed state when the specimen was unloaded. During the fatigue test, no cracks were observed on the notch-free side of the specimen.

#### 4.2.2. Comparison Study: The Amplitude of Fundamental and Second Harmonic

For the cutout structure, the complex boundary conditions result in more intricate signals, with multiple peaks frequently appearing in the amplitude plots, especially in the second harmonic. It is crucial to identify the peak amplitude *A*_2_ originating from the crack to accurately calculate *β*_CAN_, thereby enhancing crack detection. The time of flight (*t*_CAN_) of the second harmonic signal is an effective method for identifying *A*_2_ generated by the crack. Since the potential location is near the notch tip, the time of flight of the second harmonic signal from the crack can be predicted using Equation (10). As the potential location is known to be near the notch tip, one can predict the *t*_CAN_ of the second harmonic signal from the crack using Equation (10).

(10) $$t_{\mathrm{CAN}}=t_{0}+\frac{L}{2\times c_{g}^{300\mathrm{kHz}}}+\frac{L}{2\times c_{g}^{600\mathrm{kHz}}}$$

where *t*_0_ represents the time corresponding to the envelope peak of the excitation signal, $c_{g}^{300\mathrm{kHz}}$ is the group velocity at 300 kHz, $c_{g}^{600\mathrm{kHz}}$ is that at 600 kHz, and *L* is the distance between the actuator (PZT1) and sensor (PZT2). The excitation signal has its envelope peak at 16.78 μs. The modes of the detected Lamb wave can be discriminated based on *t*_CAN_ using group velocities obtained from the dispersion curves. The *t*_CAN_ values for different mode pairs are shown in Table 3. Again, it should be borne in mind that the guided wave propagates in both the 6 mm and 2 mm regions. As it travels more in the 6 mm part, the wave speed is estimated with the dispersion curves of a 6 mm plate. This is a rough estimation to help understand the experimental results. It can be seen that the second harmonic signal arriving at approximately 78 μs may contain both A0 and A1 modes. The second harmonic signal arriving at approximately 84 μs may contain the S0 mode, while the signal arriving at approximately 64.5 μs may contain the S1 mode.

**Figure 8:**
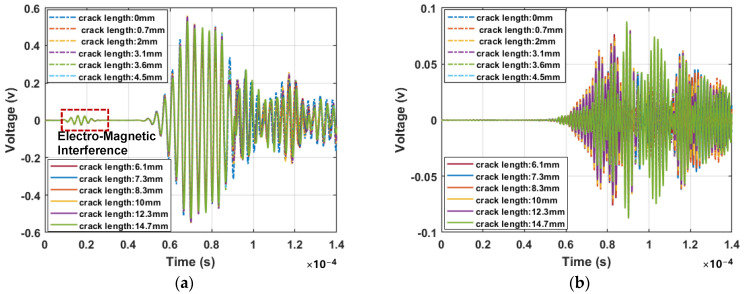
The experimental signals in sensing paths PZT1–PZT2: (**a**) 0° phase signals; and (**b**) Signals after applying phase inversion technology (mainly the second harmonics).

The experimental signals at various crack lengths, captured with the PZT1–PZT2 path, are shown in Figure 8. It can be seen that the wave packets are not separate and clear, due to the dispersion properties of the Lamb wave and boundary reflections, and the amplitude difference induced by the crack is difficult to discern directly from the time domain signals in Figure 8a. Figure 8b shows the signals of different crack lengths after applying phase inversion technology (mainly the second harmonics), from which significant amplitude variations can be observed.

**Figure 9:**
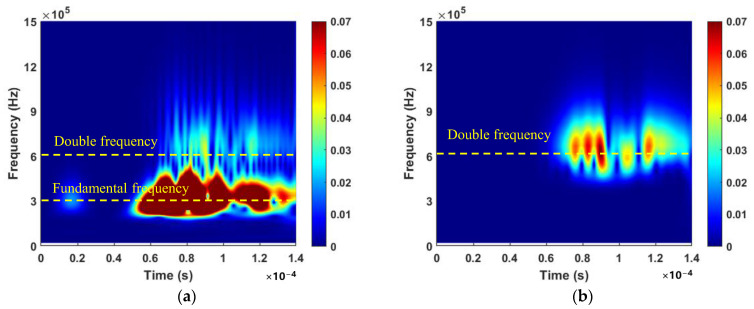
Spectra of signals at 300,000 fatigue cycles (crack length 8.3 mm). (**a**) Fundamental wave; and (**b**) Second harmonic.

**Figure 10:**
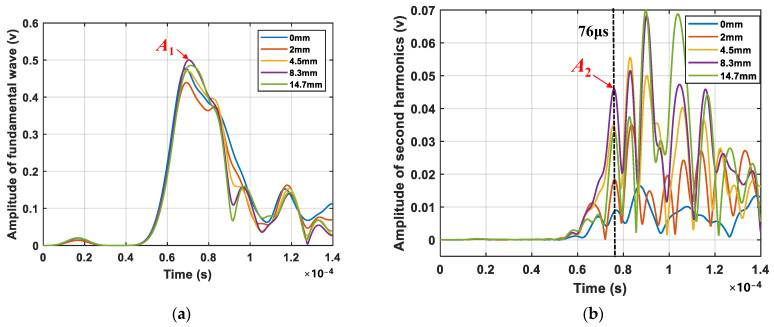
Spectral profile amplitudes for four typical crack lengths (0 mm, 2 mm, 4.5 mm, 8.3 mm, and 14.7 mm). (**a**) Fundamental wave; (**b**) Second harmonic.

Figure 9 shows the typical signal spectra captured by PZT2, which were obtained by performing a CWT on the signal (crack length 8.3 mm) from Figure 8. In Figure 9a, the time–frequency plot reveals prominent fundamental frequency components, which obscure the detection of the second harmonic. Figure 9b offers clear time–frequency information regarding the second harmonic, evidently suppressing the fundamental frequency component and enhancing the double-frequency component. The amplitude envelopes of the fundamental frequency and double-frequency components are marked by yellow dotted lines, respectively. Figure 10 illustrates the spectral profile amplitudes of the fundamental wave and second harmonics at four typical crack lengths (0 mm, 2 mm, 4.5 mm, and 8.3 mm). The maximum envelope peak, denoted by *A*_1_, is shown in Figure 10a. In Figure 10b, the spectral profile at 600 kHz reveals multiple peaks in the signal. Although the potential time of flight of the crack-induced second harmonic under four different mode pairs is shown in Table 3, significant amplitude variation patterns are only observed at 76 µs. The absence of distinct amplitude variation patterns at other times might be due to boundary reflections from the complex structure. The envelope peak at approximately 76 μs, which closely matches the calculated *t*_CAN_ of the second harmonic from the fatigue crack, suggests that the increase in the second harmonic amplitude at this location is mainly related to crack propagation. This peak is denoted as *A*_2_.

**Figure 11:**
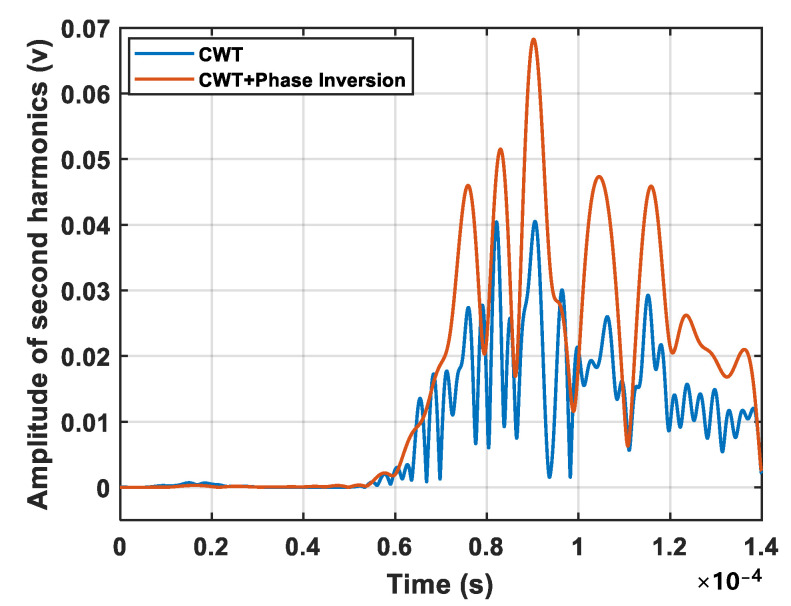
Spectral profile amplitudes of the second harmonic before and after applying the phase inversion technique for a crack of 8.3 mm.

Figure 9 also shows the benefits of the phase inversion method. Figure 11 compares the spectral profile amplitudes of the second harmonic before and after applying the phase inversion technique, i.e., the spectral amplitude at the double frequency shown in Figure 9a,b. The comparison clearly indicates a significant increase in the second harmonic amplitude and an improvement in the signal-to-noise ratio when using the phase inversion technique.

The relative differences in the spectrum amplitude of the fundamental and second harmonics are utilized as features to quantify the changes in the monitoring signals due to fatigue crack growth, respectively. They are calculated based on Equation (11).

(11) $$\Delta Amp_{j}=\frac{A_{j}(i)-A_{j}(1)}{A_{j}(1)}\times 100\%,\left(i=1,2,3\cdots 13,j=1,2\right)$$

where $\Delta Amp_{1}$ and $\Delta Amp_{2}$ are the relative changes in the fundamental and second harmonic amplitude, respectively. *A*_1_ and *A*_2_ represent the amplitude values corresponding to the fundamental and second harmonic frequencies of the monitoring signal, respectively. The index *i* = 1 to *i* = 13 denotes different crack lengths (with *i* = 1 representing 0 mm crack and *i* = 13 for 14.7 mm).

**Figure 12:**
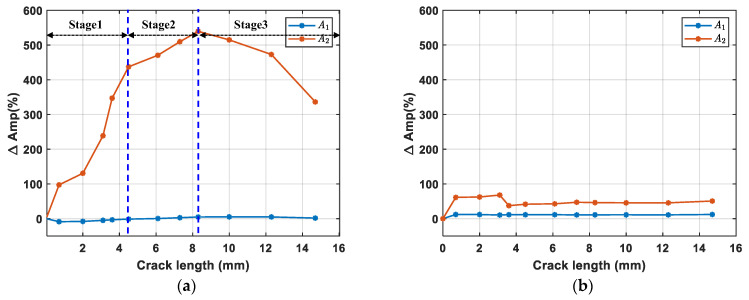
Comparison of $\Delta Amp_{1}$ and $\Delta Amp_{2}$ at crack growth. (**a**) PZT1–PZT2 (with fatigue crack); and (**b**) PZT3–PZT4 (without fatigue crack).

The relative differences in the spectral amplitude at different crack lengths are shown in Figure 12. Figure 12a shows three stages of crack propagation. During stage 1, *A*_2_ exhibits a rapid increase as the contact area expanded due to crack propagation in the 2 mm thick region. After the crack extended to the step area, a higher cyclic load is required for it to continue propagating into the thicker region. On one hand, this enlarged the total crack length, expanding the new “breathing” area; on the other hand, it tore apart the crack in the 2 mm region, reducing the contact in certain areas and consequently decreasing the “breathing” area. This led to reduced contact nonlinearity and a slower growth rate. Therefore, the growth rate of *A*_2_ begins to decelerate at stage 2. Finally, during stage 3, although the crack continued to grow, much of the crack became too large to exhibit full “breathing” behavior, so the contact area further decreased, resulting in a reduction in the amplitude of the second harmonic. Moreover, as depicted in Figure 12a, $\Delta Amp_{2}$ is more pronounced while $\Delta Amp_{1}$ shows no obvious difference with crack growth. This is due to the strong acoustic transparency of a closed crack, allowing signals to pass through the closed crack. This phenomenon demonstrates that the nonlinear component (second harmonic here) is more effective in monitoring the closed cracks compared to the linear component (fundamental wave). In addition, compared to $\Delta Amp_{2}$ in Figure 12a, Figure 12b shows no significant change in $\Delta Amp_{2}$ in the PZT3-PZT4 path during crack growth, confirming that $\Delta Amp_{2}$ is caused by the fatigue crack.

#### 4.2.3. Fatigue Crack Detection Using the Proposed Data Processing Method

**Figure 13:**
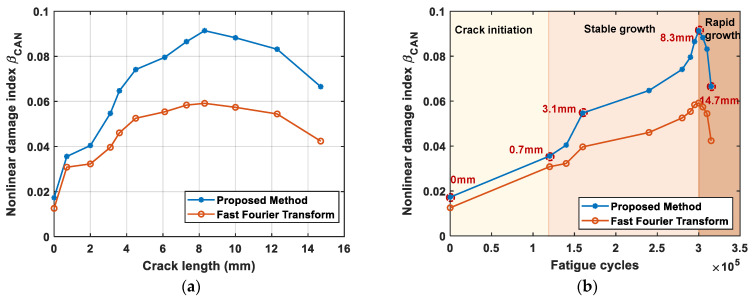
The relationship between *β*CAN and crack length (or fatigue cycles). (**a**) *β*CAN versus the crack length; and (**b**) *β*CAN versus the fatigue cycles.

With *A*_1_ and *A*_2_ obtained using the proposed method, the nonlinear index *β*_CAN_ calculated using Equation (2) can then be used to characterize the crack. The blue curve shows the relationship between *β*_CAN_ and crack length in Figure 13a, while that and fatigue cycles is shown in Figure 13b. It can be seen that the trend of *β*_CAN_ is similar to that of the amplitude of the second harmonic in Figure 13a, as there is no significant change in the amplitude of the fundamental frequency component. Figure 13b depicts the three stages corresponding to the different phases of crack growth: crack initiation, stable growth, and rapid growth. An initial value of *β*_CAN_ greater than 0 indicates the presence of a second harmonic component in the intact plate, probably from other nonlinearity, such as *β*_e_, and *β*_eq_. *β*_CAN_ increases with the progression of the fatigue crack (or loading cycles) until the crack reaches 8.3 mm, corresponding to approximately 300,000 fatigue cycles. Beyond this point, *β*_CAN_ begins to decrease due to further crack growth, resulting in the crack-opening displacement in the wake region of the crack being too large to increase the contact area of the crack surfaces, the reduction in CAN from the fatigue crack. This phenomenon is supported by recent studies that show a similar trend—a decrease in the nonlinear index during the late stages of a structure’s fatigue life [28,49,50]. This indicates that the nonlinear ultrasonic effect is sensitive to micro defects but less effective for measuring macroscopic cracks.

In order to show the effectiveness of the proposed method, *β*_CAN_ calculated directly from the fast Fourier transform (FFT) of the original signal form, as the ratio of the magnitude of the double frequency and fundamental frequency, is plotted in orange in Figure 13. It can be seen that the proposed method demonstrates a significantly higher sensitivity to crack length variations. As the crack length increases, the nonlinear damage index for the proposed method rises sharply, reaching a peak around 8 mm, indicating strong responsiveness to crack growth. In contrast, the FFT method shows much less sensitivity, with its damage index remaining relatively flat across most crack lengths. While the sensitivity of the proposed method decreases slightly after 8 mm, it continues to be more responsive than the FFT method. This indicates that the proposed method is more effective at detecting crack growth compared to the FFT.

The above analysis indicates that *β*_CAN_ calculated through the proposed method can effectively characterize the fatigue crack growth (or fatigue cycles) in a typical aircraft component with a relatively complex structural form.

## 5. Conclusions

To effectively monitor the fatigue cracks in aerospace metal structures with varying thickness and large openings, this paper proposes an integrated data processing method that combines phase inversion and a Morlet wavelet-based CWT algorithm. The Lamb mode pair is selected with phase velocity mismatches between the primary wave and second harmonic to minimize the accumulation of distributed material nonlinearity in the structure. Consequently, the nonlinear components in the sensing signal can be primarily attributed to the nonlinearity generated at the fatigue crack. The principal findings of this research are summarized as follows:

(1) During fatigue crack propagation, the crack remains closed when the specimen is unloaded. $\Delta Amp_{1}$ shows no significant variation due to the high acoustic transparency of closed cracks, while $\Delta Amp_{2}$ exhibits a distinct trend of the initial oscillating increase, and then subsequently decreases with crack growth. The result confirms that the nonlinear component (second harmonic, here) is more effective than the linear component (fundamental wave) for monitoring closed cracks.
(2) The proposed integrated data processing method effectively extracts second harmonic components in sensing signal, identifies the envelope peak *A*_2_ of the second harmonic generated at the fatigue crack, and then calculates the nonlinear parameter *β*_CAN_. The parameter first increases monotonically with crack growth and then decreases after the crack reaches 8.3 mm due to the crack becoming too large to exhibit a fully ‘breathing’ behavior. Experimental results validate the efficacy of the proposed method in detecting fatigue crack growth in complex structures.

## Figures and Tables

**Table 1:** Material parameters of the Al6061 aluminum alloy.

Density (kg/m^3^)	Elastic Modulus (Gpa)	Poisson’s Ratio
2.78	70	0.33

**Table 2:** The relative deviation and the maximum cumulative propagation distance.

Mode Pair	$c_{\mathrm{ph}}^{300\mathrm{kHz}}$ (m/s)	$c_{\mathrm{ph}}^{600\mathrm{kHz}}$ (m/s)	*D*	*L*_max_ (mm)
S0-A0	4875	2801	42.54%	5.49
S0-S0	4875	3024	37.97%	6.64
S0-A1	4875	5165	5.90%	72.35
S0-S1	4875	6082	24.7%	20.47

**Table 3:** The theoretically calculated *t*_CAN_.

Mode Pair	$c_{g}^{300\mathrm{kHz}}$ (m/s)	$c_{g}^{600\mathrm{kHz}}$ (m/s)	*t*_CAN_ (μs)
S0–A0	3672	2981	77.56
S0–S0	3672	2472	84.47
S0–A1	3672	2952	77.89
S0–S1	3672	4385	66.81

## Data Availability

The data presented in this study are available on request from the corresponding author. (The data are not publicly available due to privacy).
